# The symbiotic bacteria *Alcaligenes faecalis* of the entomopathogenic nematodes *Oscheius* spp. exhibit potential biocontrol of plant‐ and entomopathogenic fungi

**DOI:** 10.1111/1751-7915.13365

**Published:** 2019-01-07

**Authors:** Shaojie Shan, Wenwu Wang, Chunxu Song, Minggang Wang, Bingjiao Sun, Yang Li, Yaqi Fu, Xinghui Gu, Weibin Ruan, Sergio Rasmann

**Affiliations:** ^1^ College of Life Sciences Nankai University Tianjin 300071 China; ^2^ Department of Molecular Genetics Groningen Biomolecular Sciences and Biotechnology Institute University of Groningen Groningen The Netherlands; ^3^ Department of Plant Protection Biology Swedish University of Agricultural Sciences PO Box 102 SE‐23053 Alnarp Sweden; ^4^ Disease and Insect Bio‐control Engineering Research Center of National Tobacco Industry Yuxi 653100 Yunnan China; ^5^ Laboratory of Animal Ecology and Entomology Institute of Zoology University of Neuchâtel CP 2 CH‐2007 Neuchâtel Switzerland

## Abstract

Soil‐dwelling entomopathogenic nematodes (EPNs) kill arthropod hosts by injecting their symbiotic bacteria into the host hemolymph and feed on the bacteria and the tissue of the dying host for several generations cycles until the arthropod cadaver is completely depleted. The EPN–bacteria–arthropod cadaver complex represents a rich energy source for the surrounding opportunistic soil fungal biota and other competitors. We hypothesized that EPNs need to protect their food source until depletion and that the EPN symbiotic bacteria produce volatile and non‐volatile exudations that deter different soil fungal groups in the soil. We isolated the symbiotic bacteria species (*Alcaligenes faecalis*) from the EPN *Oscheius* spp. and ran infectivity bioassays against entomopathogenic fungi (EPF) as well as against plant pathogenic fungi (PPF). We found that both volatile and non‐volatile symbiotic bacterial exudations had negative effects on both EPF and PPF. Such deterrent function on functionally different fungal strains suggests a common mode of action of *A. faecalis* bacterial exudates, which has the potential to influence the structure of soil microbial communities, and could be integrated into pest management programs for increasing crop protection against fungal pathogens.

## Introduction

Soil‐dwelling nematodes (phylum Nematoda) occupy a large fraction of the soil's living matter and are key biotic agents influencing several ecosystem functions such as ecosystem productivity, soil organic matter decomposition and nutrient cycling (Kaya and Gaugler, 1993; Williamson and Gleason, 2003; Yeates *et al*., 2009; Kergunteuil *et al*., 2016). Among soil nematodes, entomopathogenic nematodes (EPNs), which are obligate predators of arthropods, are widely studied because of their efficiency in controlling arthropod pests of crops (Poinar, 1990) in a variety of agro‐ecosystems (Shapiro‐Ilan *et al*., 2006; Heve *et al*., 2016). While most of the EPNs are known belonging to the genera *Heterorhabditis* and *Steinernema*, recently, *Oscheius* spp. was also found to be entomopathogenic, carrying several bacteria species (Park *et al*., 2011). The mode of infection of EPNs in commercial can be summarized as follows. First, the third‐instar, free‐living, infective juveniles (IJs) enter the arthropod host through the mouth, spiracles and anus. Second, the nematodes release symbiotic bacteria from the intestines into the insect hemolymph. Third, the bacteria multiply rapidly and kill the host within 48 h, while the young nematodes feast on the bacteria and the tissue of the dying insect, grow and reproduce within the dead host (Wang and Gaugler, 1998). When the host cadaver is depleted of resources, third instar EPNs and their symbiotic bacteria emerge as the next generation of infective juveniles (IJs) in the soil, ready for searching and entering a novel host (Poinar, 1990).

The EPN–bacteria–arthropod cadaver complex, in turn, can also serve as energy source for the surrounding soil biota. For instance, entomopathogenic fungi (Resquín‐Romero *et al*., 2016), entomopathogenic bacteria (Mishra, 2018), predatory insects (Zhou *et al*., 2002; Fenton *et al*., 2011) and other free‐living nematodes (Campos‐Herrera *et al*., 2015) could all take advantage of this newly formed energy source in the soil. Therefore, to be successful and evolutionary stable, the EPN–bacteria mutualism should put in place strategies to prevent the attack from all those other opportunistic scavengers. Non‐volatile and volatile metabolites have been shown to play a crucial role in mediating interactions within the soil ecosystem (Rasmann and Turlings, 2016; van Dam and Bouwmeester, 2016). For example, non‐volatile root exudates have been shown to shape soil fungal community composition and diversity (Broeckling *et al*., 2008). Similarly, volatile organic compounds have been shown to mediate a multitude of interactions among plant and other soil biota, or among soil biota (Weisskopf *et al*., 2016; Bailly and Weisskopf, 2017). These volatiles can promote plant root development (Bailly *et al*., 2014) or defend plants against infection by root pathogens (Sohrabi *et al*., 2017). Furthermore, these volatiles can also be used by EPNs as cues to locate their hosts near the site of root wounding (Rasmann *et al*., 2005; Ali *et al*., 2010). Interestingly, it was shown that EPNs‐symbiotic bacteria are constantly releasing volatile and non‐volatile metabolites into soil (Jones *et al*., 2017), therefore potentially mediating biotic interactions.

We here hypothesized that EPN‐symbiotic entomopathogenic bacteria volatile and non‐volatile exudations can act as deterrent against a wide range of soil microbes. Specifically, we asked whether the exudation of EPN‐mutualistic bacteria could affect the growth of (i) entomopathogenic fungi (EPF) and (ii) plant pathogenic fungi (PPF). By testing different fungal guilds of the soil, we aimed at detecting a generalizable activity of bacterial exudation on the surrounding fungal community. We designed a series of infectivity bioassays using symbiotic bacteria from field‐isolated EPNs. We ran these bioassays adding non‐volatile and volatile bacteria exudates and analysed the active compounds in these chemicals. If entomopathogenic bacteria could deter entomopathogenic fungi, this could contribute maintaining entomopathogenic nematode populations, and in turn favour their control effect on arthropod pests of plants. If entomopathogenic bacterial could deter plant‐pathogenic fungi, this could directly favour plant performance (Fig. S1).

## Results

### Antifungal effect of entomopathogenic bacteria non‐volatile compounds

#### Petri dish experiment

**Figure 1:**
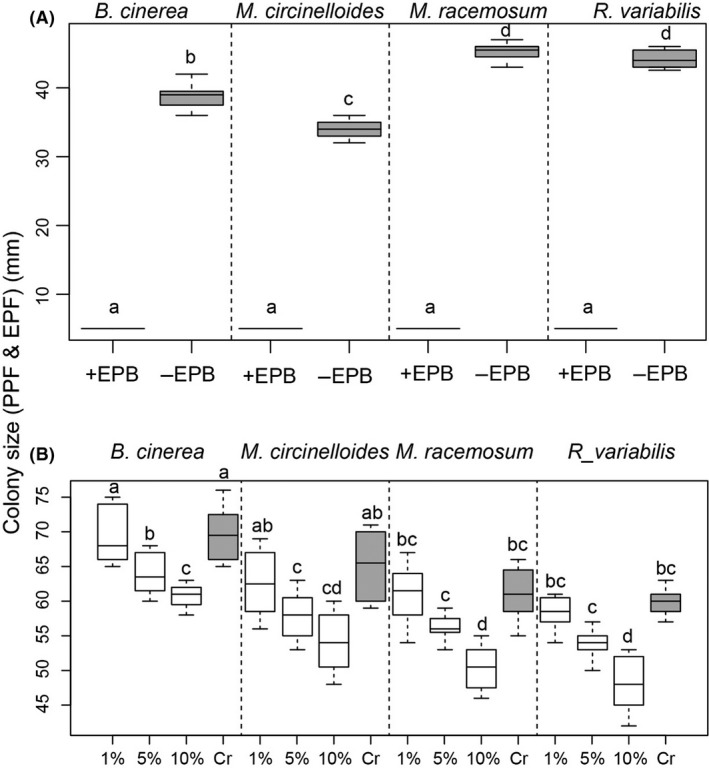
Colony development (shown as the boxplots of average colony diameter in mm) of PPF *Botrytis cinerea*, and EPF *Mucor circinelloides*,* M. racemosus* and *Rhizomucor variabilis* in the presence (+EPB), or the absence (−EPB) of *Alcaligenes faecalis*. Panel (A) shows the effect using whole cell cultures. (Panel B) represents fungal colony developments when in presence of EPB cell‐free filtrates (1%, 5% and 10% dilution of the initial filtrate. Grey boxes represent the control without EPBs. Letters above boxplots indicate significant difference among treatments and species (*P *≤* *0.05, Turkey HSD).

We found that in the presence of EPB *A. faecalis*, the colony size of all three EPFs did not change from the initial size of 5 mm in diameter, while in the absence of EPB, the colony size increased dramatically for all fungal species to an average of about 40 mm in diameter (Fig. 1A, treatment effect, *F*_1,87_ = 33125, *P *<* *0.0001). The size of the colony differed among EPF species (Fig. 1A, species effect, *F*_3,87_ = 181, *P *<* *0.0001, and species × treatment effect, *F*_3,87_ = 181, *P *<* *0.0001). The cell‐free experiment corroborated the above results and showed a strong inhibitory effect of the EPB filtrate on fungal colony size, but this was dose dependent. Only the 5% and 10% dilutions showed a significant inhibition of the fungal colony (Fig. 1B, treatment effect, *F*_3,87_ = 263, *P *<* *0 .0001). The size of the colony differed among EPF species (Fig. 1B, species effect, *F*_3,87_ = 2524, *P *<* *0.0001, and species × treatment effect, *F*_3,87_ = 1.52, *P *=* *0.14).

#### Cherry tomato fruit experiment

**Figure 2:**
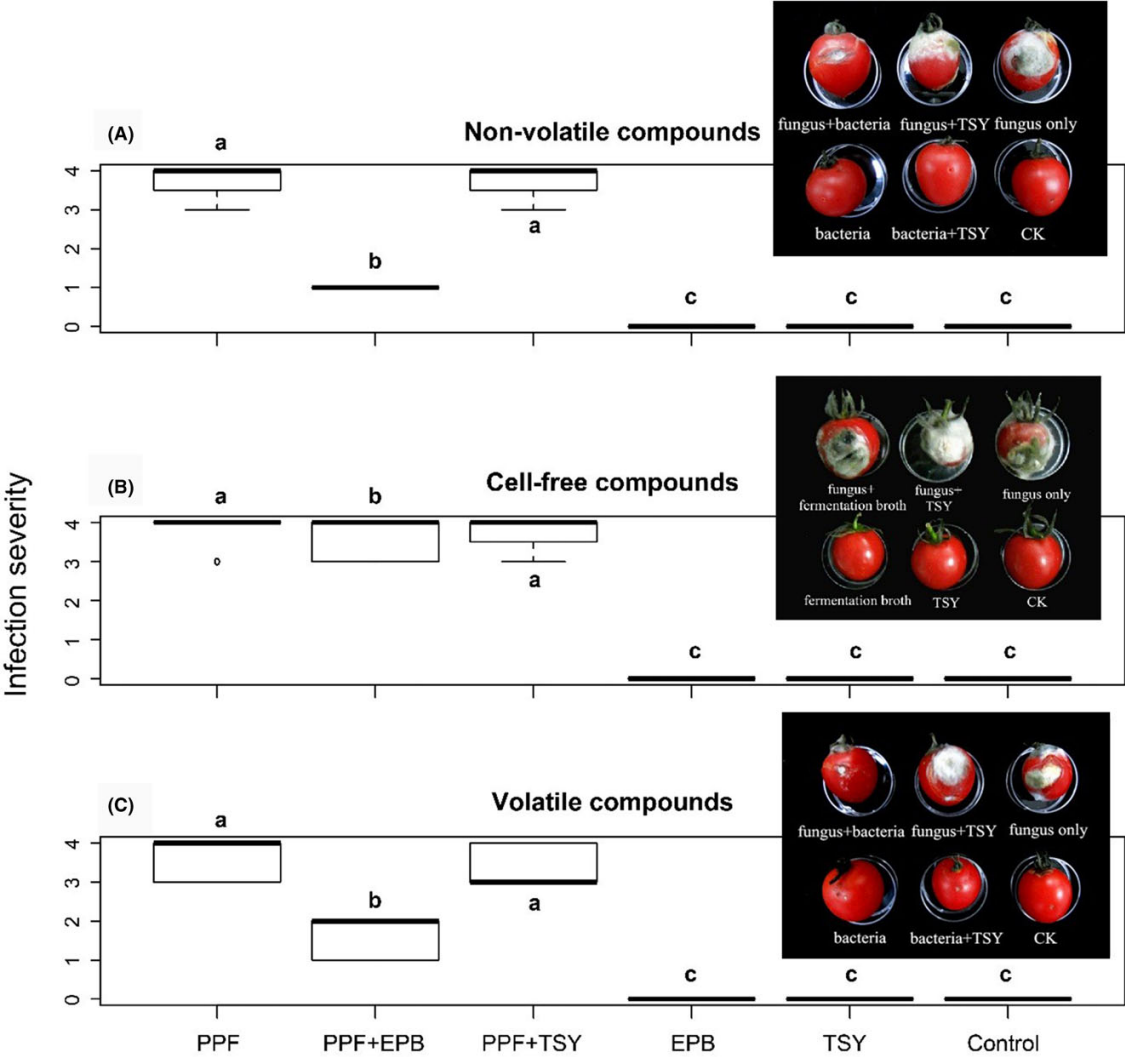
Inhibition of PPF *Botrytis cinerea* growth by metabolites of EPB *Alcaligenes faecalis*. Shown are boxplot for disease infection severity due to PPF on tomato fruits under (A) direct contact, (B) using cell‐free filtrates and (C) transmission of volatile organic compounds. EPB: symbiotic bacteria; PPF: plant pathogenic fungi; TSY: TSY medium; Control: sterile distilled water. After 7 days inoculation, the severity of infections was determined as the proportion of decayed area according to a scale from 0–4: 0, No decay; 1, decayed area < 1/8; 2, decayed area from 1/8 to 1/4; 3, decayed area from 1/4 to 1/2; 4, decayed area > 1/2. An example of infection severity is shown in the inserted figure in the graph.

We found a strong inhibitory effect of EPB *A. faecalis* on the development of the PPF *B. cinerea* on tomato fruits, in which damage severity dropped three points in average when the PPF were co‐inoculated with EPBs. (Fig. 2A, treatment effect, *F*_66,205_ = 601, *P *<* *0.001). We also found an inhibitory effect of *A. faecalis* cell‐free supernatant on the development of the PPF *B. cinerea* on tomato fruits, but to a lesser extent than when compared to the full cellular extract (Fig. 2B, treatment effect, *F*_66,205_ = 601, *P *<* *0.001).

#### In vivo antifungal effects of symbiotic bacteria against EPF on *G. mellonella* larvae

The effect of the EPB on colonization by three EPF on *G. mellonella* larvae was dramatic. None of the larvae that were inoculated with EPB showed signs of fungal infection after 4 days, while all larvae that did not receive EPB resulted to be infected, independently of the sterile water or TSY medium treatment application (Fig. S3).

### Antifungal effect of entomopathogenic bacteria volatile organic compounds

#### Petri dish experiment

**Figure 3:**
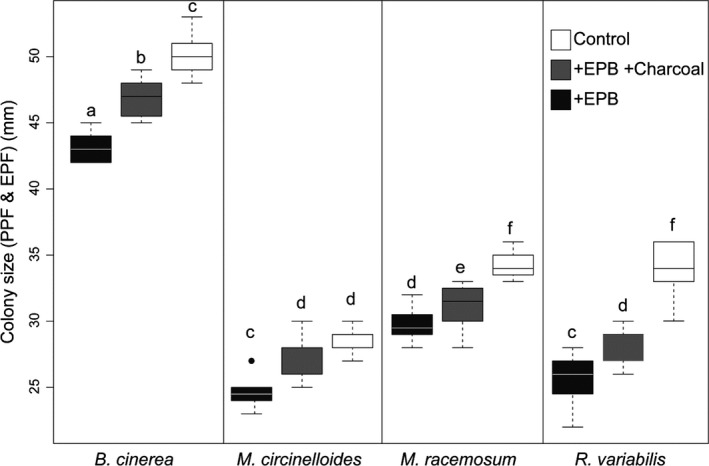
Colony development (shown as the boxplots of average colony diameter in mm) of the PPF *Botrytis cinerea*, and the EPF *Mucor circinelloides*,* M. racemosus* and *Rhizomucor variabilis* in the presence of (i) EPB *Alcaligens faecalis* (black boxes, +EPB), (ii) in the presence of *A. faecalis* and charcoal in the chamber (+EPB + Charcoal) and (iii) or the absence of *A. faecalis* bacterial colonies producing volatile organic compounds in the chamber (−EPB). Letters above boxplots indicate significant differences among treatments and species (*P *≤* *0.05, Turkey HSD).

Volatiles released by EPB *A. faecalis* significantly inhibited the growth of PPF *B. cinerea* (Fig. 3, treatment effect, *F*_2,131_ = 228, *P *<* *0.001), for all four fungal species, although with some degree of variation across species (species effect, *F*_3,131_ = 1523, *P *<* *0.001, and species × treatment effect, *F*_6,131_ = 8.47, *P *<* *0.001).

#### Cherry tomato fruit experiment

We found a strong inhibitory effect of EPB *A. faecalis* VOCs on the development of the PPF *B. cinerea* on tomato fruits (Fig. 2C, treatment effect, *F*_65,66_ = 281.51, *P* < 0.001).

#### EPB VOCs collection and analysis

Chromatographic analyses of VOCs produced by the EPB *A. faecalis* (using the 75 μm Carboxen/PDMS fibre, which displayed the best extraction efficiency) showed that dimethyl disulfide was the most abundant compound produced (32%), followed by isobutyl isovalerate (5%; Table 1, Fig. S6).

**Table 1:** Volatile organic compounds profiles of EPB *Alcaligenes faecalis*. Shown are the retention times, and the relative peak area compared to the total area of each compound sampled using a 75 μm Carboxen/PDMS SPME fibre

Retention time (min)	Compound	CAS	Relative area (%)
3.989	Dimethyl disulfide	000624‐92‐0	31.654
8.345	1,3‐xylene	000108‐38‐3	3.701
9.258	1,3,5,7‐cyclooctatetraene	000629‐20‐9	2.393
10.390	Butyl isobutyrate	000097‐87‐0	3.385
12.456	Dimethyl trisulfide	003658‐80‐8	1.962
14.288	Isobutyl isovalerate	000589‐59‐3	4.904
18.052	Butanoic acid, 2‐methyl‐, 3‐methylbutyl ester	027625‐35‐0	0.353
18.260	Isopentyl isopentanoate	000659‐70‐1	1.709
18.362	Butanoic acid, 3‐methyl‐, pentyl ester	025415‐62‐7	3.859
19.598	(1r,4r)‐(+)‐camphor	000464‐49‐3	0.328

#### Antifungal effects of pure VOCs compounds against PPF and EPF

**Figure 4:**
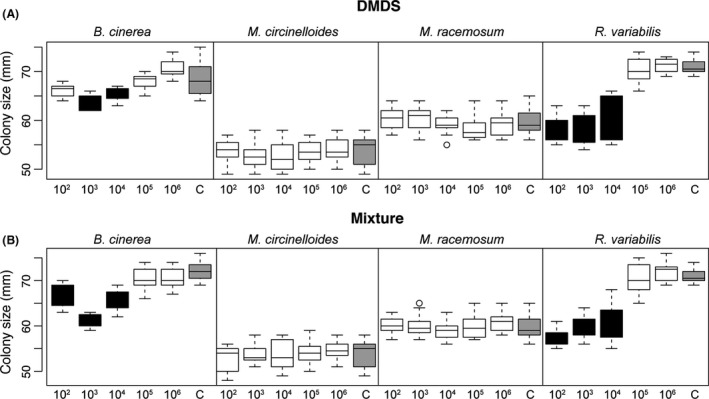
Antifungal activity of dimethyl disulfide (DMDS) and a mixture of pure compounds against PPF *Botrytis cinerea,* and EPF *Mucor circinelloides*,* M. racemosus* and *Rhizomucor variabilis*. Shown are the boxplots for (A) the effect of a serial dilution (10^2^, 10^3^, 10^4^, 10^5^, 10^6^) of dimethyl disulfide (DMDS) and (B) effect of the same serial dilution of a compound mixture. For the mixture, the compounds were mixed according to the ratio: dimethyl disulfide : isobutyl isovalerate : isopentyl isopentanoate = 20 : 4 : 1 (m:m:m). Control corresponds to no compounds added to the chamber (grey boxes). Black boxes show significant difference compared to the control (*P *≤* *0.05, Turkey HSD).

We found that, both for the DMDS and mixture of VOCs experiments, a variable effect of serial dilution of EBP *A. faecalis* on the colony size of different fungal species [Fig. 4A (DMDS), serial dilution treatment effect, *F*_5,263_ = 48.79, *P *<* *0.001, species effect, *F*_3,263_ = 556.8, *P *<* *0.001 and species × treatment effect, *F*_15,263_ = 25.18, *P *<* *0.001. Figure 4B (Mixture), serial dilution treatment effect, *F*_5,263_ = 79.19, *P *<* *0.001, species effect, *F*_3,263_ = 608.9, *P *<* *0.001 and species × treatment effect, *F*_15,263_ = 28.08, *P *<* *0.001]. Specifically, only *B. cinerea* (the PPF) and the EPF *R. variabilis* responded positively to the pure compound application, but only on dilutions lower than 10^5^ (see black boxes in Fig. 4). We did not detect antifungal activity for the isopentyl isopentanoate and isobutyl isovalerate (Fig. S8).

## Discussion

We isolated the symbiotic bacterium (EPB) *A. faecalis* from the entomopathogenic nematode *Oechinus* spp. from the field and ran bioassays against entomopathogenic fungi (EPFs) and plant‐pathogenic fungi (PPF) by separating non‐volatile and volatile bacterial exudates. We found that the exudates of the symbiotic bacteria had negative effects on both PPF and EPF.

### Entomopathogenic bacteria and their association with entomopathogenic nematodes

Besides the classically studied EPN species in the genera *Steinernema* and *Heterorhabditis*, several nematode species from genus *Oscheius* have been confirmed as EPNs (Ye *et al*., 2010; Pervez *et al*., 2013; Zhou *et al*., 2017). Similar to *Steinernema* and *Heterorhabditis*,* Oscheius* species are in association with several bacteria strains that belong to three classes; namely the beta‐proteobacteria (*Alcaligenes*), the gamma‐proteobacteria (*Enterobacter, Proteus, Providencia, Pseudomonas, Stenotrophomonas*) and the Bacilli (*Bacillus, Enterococcus, Lysinibacillus*; Sangeetha *et al*., 2016). In our experiment, we primarily isolated the bacterium *A. faecalis;* however, this does not exclude *Oscheius* nematodes to bear other bacteria species. Indeed the isolation method we choose, based on the selectivity for bromothymol blue, favours the isolation of specific bacterial variants, in our case *A. faecalis*.

While the general dogma suggests that *Steinernema* and *Heterorhabditis* are in association with unique bacterial strains (Forst *et al*., 1997; Goodrichblair, 2007; Clarke, 2008), *A. faecalis* strain MOR02 was also found in the oesophagus and intestine of the *Steinernema feltiae*,* S. carpocapsae* and *H. bacteriophora*. Later, this *A. faecalis* strain was confirmed to be an entomopathogenic bacterium; *A. feacalis* MOR02‐inoculated *G. mellonella* larvae suffered more than 96% mortality after 24 h (Quiroz‐Castañeda *et al*., 2015). Therefore, *A. faecalis* can cause mortality of arthropod larvae via haemocoelic lesion and can be used as food source for EPN growth, development and reproduction (Park *et al*., 2011).

Recently, it has been suggested that EPNs could kill an arthropod host without the use of symbiotic bacteria. For instance, the EPN *S. carpocapsae* was shown to be able to kill insects and partly degrade insect tissues in the absence of any bacteria; however, the pathogenicity of such axenic nematodes was weaker than that of monoxenic nematodes (Darsouei *et al*., 2017). While, axenic *H. bacteriophora* were unable to kill *G. mellonella* larvae, axenic *S. carpocapsae* could kill insect hosts but produce very low number of progeny (Han and Ehlers, 2000). This together suggests an almost obligatory relationship between EPNs and their symbionts. Indeed symbiotic bacteria are also able to suppress the immune system of the hosts (Darsouei *et al*., 2017), thus favouring nematode colonization of their host and successful propagation. Future research should thus highlight the specific impact of *A. faecalis* on the hosts’ immune responses, and ultimately the fitness benefits for *Oscheius* nematodes.

To summarize, a multitude of different bacterial species can co‐exist inside a nematode (Berg *et al*., 2016). Recently, Maher *et al*. (2016) found that EPNs could extend their ecological niche breadth by associating with different symbiotic bacteria. Therefore, the spectrum of ecological consequences that are mediated by EPB extends from influencing the surrounding soil biota to modifying the behaviour of the symbiotic nematodes (Kenney and Eleftherianos, 2015), which should cascade down to influence the whole soil community dynamics of interactions.

### EPBs protect infected host cadavers

In the present study, volatile and non‐volatile metabolites of EPB *A. faecalis* showed antifungal activity against the entomopathogenic fungi, *M. circinelloides*,* M. racemosus* and *R. variabilis*, all isolated from the same area in which the EPN *Oscheius* sp. naturally occurred. All together, this confirms that *A. faecalis* acts as an EPB that could benefit EPNs against opportunistic soil‐dwelling entomopathogenic fungi when in association with an arthropod host cadaver. Generally, an EPN infects arthropods and then releases symbiotic bacteria into the hemolymph, which induce septicemia that kills the host, rendering the insect host corpse suitable for EPN development and reproduction. Depending on the available resources, one or more EPN generations will occur within the cadaver, and a large number of infective juveniles are gradually released into environment for infecting other hosts and continuing their life cycle (Poinar, 1990). Since the defence system of the insect cadavers is completely lost, the symbiotic bacteria must then provide defences in order to avoid the loss of the EPN population inside the cadaver (Jones *et al*., 2016) from foraging insects ants (Zhou *et al*., 2012), cricket and wasps (Gulcu *et al*., 2012), beetles (Jones *et al*., 2017), bird (Fenton *et al*., 2011) and even fishes (Raja *et al*., 2016). However, besides the abovementioned predators, insect cadavers containing EPNs will also suffer risk of infection from entomopathogenic fungi, entomopathogenic bacteria and even competition with other soil free‐living nematodes. While we here did not address the nature of non‐volatile metabolites released by *A. faecalis* that increase insect host cadaver resistance against EPF, we were able to detect a variety of volatile organic compounds produced by *A. faecalis*, including dimethyl disulfide (DMDS), toxic to EPF. Specifically, we found that pure DMDS and mixture of main components of volatile metabolites of *A. faecalis* had negative effects on the EPF *R. variabilis* in a dose‐dependent manner. Several *A. faecalis* VOCs have been previously characterized. For instance, Bhattacharyya *et al*. (2015) identified several VOCs from *A. faecalis* including butyric acid, propionic acid and benzoic acid, and reported that those VOCs induced tolerance to salt stress in *Arabidopsis thaliana* by influencing the auxin and gibberellin pathways and upregulating the expression of key ion transporters (Bhattacharyya and Lee, 2017). In contrast to soluble compounds, volatile organic compounds can diffuse easily through air‐ and gas‐filled pores in the soil and likely play an important role in long‐distance microbial interactions. Our results in combination with Bhattacharyya's research thus suggest that *A. faecalis* VOCs can influence a wider range of ecological relationships. Therefore, the ecological function of *A. faecalis* chemical exudates has to be studied on a case‐by‐case in order to understand the consequences of each individual compound on the soil community interactions.

### EPBs protect plants against EPF and insect pests

Based on our results, we argue that EPBs can favour plant in two ways: first, in our study, *A. faecalis* non‐volatile and volatile metabolites, such as DMDS, inhibited the growth of the plant pathogenic fungi *B. cinerea*. This is in line with studies showing that volatile compounds such as DMDS carry antifungal properties against a wide range of PPF (Pecchia *et al*., 2017). Interestingly, *R. variabilis* EPF has been reported to act as entomopathogenic, as well as being pathogenic to plants (Kulimushi *et al*., 2017), demonstrating the breadth of potential effects of *A. faecalis* exudates on the surrounding fungal community.

Second, we argue that EPBs can attract and maintain EPNs near the pest cadaver, thus potentially maintaining viable populations of EPNs near the rhizosphere. It was previously shown that EPNs can use root‐derived volatiles, including terpene compounds but also DMDS (Turlings *et al*., 2012), for locating their host (Stock *et al*., 2005; Tonelli *et al*., 2016). Therefore, we could speculate that the continuous presence of cadavers in the root zone, and the subsequent continuous release of volatiles, might exert a positive effect on EPNs populations, in turn favouring plants by decreasing arthropod attack and by had potential negative effect on fungal infection.

### Implementing EPB into IPM strategies

Different combinations of EPNs and commercial EPF for increasing plant growth and protection have already been attempted in several greenhouse and field trials. Generally, however, they did not show clear additive or synergistic effect of both biocontrol agents and even find negative interactive effects (Wu *et al*., 2014; Imperiali *et al*., 2017; Wakil *et al*., 2017). EPNs were detected in larvae only in a few cases (11%) in the EPF and EPNs combination treatments (Tarasco *et al*., 2011). In another study, insect larvae pre‐inoculated with the EPF *Beauveria bassiana* prior to nematode application yielded substantially lower number of IJs indicating apparent antagonistic interaction between EPF and EPN within the insect (Darissa and Iraki, 2014). Because of such potentially strong nematicidal activity (Hao Lu *et al*., 2014), *A. faecalis* might not be best to directly use in combination with EPNs without additional research. For instance, due to its insecticidal character (Essawy *et al*., 2015), DMDS has been recommended as a more ecologically sound alternative to the very polluting CH_3_Br for soil fumigations. Further studies should thus be put forward for integrating high DMDS production *A. faecalis* strains into integrated pest management control programs. Applying EPNs with such high DMDS‐producing strains in the field will reduce both fungal and arthropod pest load near roots of crop plants (Yokoyama *et al*., 2013), ultimately helping reducing synthetic pesticide application worldwide.

## Experimental procedures

### Isolation and identification of microorganisms

The field site used for collecting entomopathogenic nematodes (EPNs) and symbiotic microbial communities (entomopathogenic bacteria EPB, and entomopathogenic fungi EPF) is situated in Yuxi, Yunnan province, China (24.17291°N; 102.524987°E). Sampling occurred during summer 2016. Five soil cores of about 20 cm depth were collected using 3.6 cm diameter augers, and bulked into a single sample of about 1.5 Kg of soil, mixed thoroughly and placed into a cooler for transport.

#### Entomopathogenic nematodes (EPNs)

Collection of EPNs was done by placing five final instars of *Galleria mellonella* (Lepidoptera: Pyralidae) larvae in plastic cups (top diameter 7.5 cm, bottom diameter 4.8 cm, height 8 cm) filled with 180 g of fresh soil, about 10% moisture, and kept at 25°C. After 5 days, all dead larvae were removed from the soil in the cups and placed on White traps (White, 1927) for EPN emergence. The emerged third‐instars juvenile EPNs were rinsed three times with sterile distilled water and stored at 14°C. To double check for their pathogenicity against *G. mellonella* larvae, the recovered EPN juveniles were again added to a 6 cm petri dish filled with a sterile sand (about 10% moisture) containing with 5 *G. mellonella* larvae. The subsequent isolation of EPNs followed the same protocol as above. Final EPN colonies were stored at 16°C for further use. For taxonomic assignment, EPNs’ DNA was extracted from a single female adult nematode, which was dissected to remove bacterial cells, and placed into a PCR tube with 10 μl of lysis buffer (50 mM KCl, 10 mM Tris pH 8.3, 2.5 mM MgCl_2_ · 6H_2_O, 0.45% Nonidet P‐40, 1% Triton X‐100 and 60 μg ml^−1^ proteinase K). The mixture was incubated at 60°C for 1 h and 95°C for 15 min in a thermocycler, for amplification and sequencing (see supplementary methods in Appendix S1). Based on the sequence comparison using blast search at Genebank, EPNs were identified as *Oscheius* sp. (Rhabditida: Rhabditidae).

#### Entomopathogenic bacteria (EPB)

EPB were obtained after inoculating last‐instars *G. mellonella* larvae with 15 μl of nematode suspension (approximately 100 IJs per larvae) in a 24‐well cell culture plate lined with filter paper. Infected larvae were maintained at 25°C for 36–48 h, after which, all dead larvae were surface‐sterilized with 75% alcohol for 1 min. A drop of haemolymph was next added onto NBTA medium (45 g nutrient agar, 25 mg bromothymol blue and 40 mg triphenyl tetrazolium in 1 l of distilled water) in a 9 cm Petri dishes and incubated at 25°C. After 48 h, pure colonies of the bacteria were inoculated on nutrient agar medium and incubated at 25°C for 48 h. The bacterial isolates were cultured in trypticase soy yeast (TSY) medium (4% tryptic soy broth, 0.5% yeast extract) and finally stored at 4°C until experiments (see below). EPB DNA extraction was performed using Bacteria Genomic DNA Kit (CWBiotech, Beijing, China). EPB was grown in TSY medium at 22°C, 150 rpm for 36 h in an incubator shaker (HNY‐2102C; Tianjin Honour Instrument Co. Ltd., Tianjin, China). Based on the sequence comparison using blast search at Genebank (Appendix S1), the EPB identified was the Gram‐negative bacteria *Alcaligenes faecalis* (Burkholderiales: Alcaligenaceae).

#### Confirmation of bacterial symbionts

To confirm that the collected EPBs were in the nematode gut and not on the surface, we performed an additional nematode surface sterilization experiment based on Berg *et al*. (2016). Specifically, nematodes were collected in a 50 ml tube, transferred to a 15 ml conical tube and washed six times with M9 salt solution (Sigma‐Aldrich, Saint Louis, MO, USA) to remove soil particles and external bacteria. In each wash, worms were either allowed to precipitate by gravity (to separate adults from larvae) or centrifuged at 1800 rpm for 3 min, supernatant was removed, and the pellet was re‐diluted (100‐fold volume of M9 added). Following washes, worms were placed onto an un‐seeded NGM plate supplemented with 100 μg ml^−1^ gentamicin for 1 h for surface sterilization. A sample of the last wash medium was spread on an LB plate without antibiotics to confirm lack of external bacteria. The washed and surface‐sterilized worms were ground using a motorized pestle in 300 μl of M9, pelleted, and bacteria from the grown on medium (25°C, 2 days). Isolates were identified through sequencing of the full‐length 16S rDNA gene as described above and yielded to the same *A. faecalis* species.

#### Entomopathogenic fungi (EPF)

For EPF extraction, 20 g of soil were added with 1 ml of distilled water and placed in a 9 cm diameter Petri dish. Two last‐instars *G. mellonella* larvae were then added to each dish and incubated at 27°C until larvae died. The fungi growing on the larval cadavers were next inoculated on potato dextrose agar (PDA) medium, and the purified fungal colony was then re‐inoculated onto additional *G. mellonella* larvae for two complete culture cycles to ascertain pathogenicity (i.e. the purified culture killed the larvae). During each culture cycle, a Petri dish without the fungal addition was used as control. The confirmed fungal pathogens were rDNA sequenced (Appendix S1). Fungal DNA was extracted using DNA quick Plant System (Tiangen Biotech Ltd, Beijing, China). Based on the sequence comparison using blast search at Genebank, three species of EPF were identified as *Mucor circinelloides*,* Mucor racemosus* and *Rhizomucor variabilis* (Mucoraceae). The isolated fungal colonies were stored at 4°C on PDA medium (potato‐glucose‐agar) and routinely subcultured on PDA (pH 6.50) in Petri dishes (90 mm in diameter) in dark at 25°C.

#### Experimental design

We divided the experiments in two parts, first to test the direct effect of EPB *A. faecalis* bacteria non‐volatiles metabolites on plant pathogenic (PPF) and on entomopathogenic fungi (EPF), and second, to test for the effect of EPBs volatile organic compounds (VOCs) on the same combinations of microbes. For EPF, we used the soil‐isolated (*Mucor circinelloides*,* Mucor racemosus*,* Rhizomucor variabilis*). For PPF, we used *Botrytis cinerea* (Helotiales, Sclerotiniaceae), which was kindly provided by Yonghong Li, Nankai University and maintained on PDA medium.

### Antifungal effect of entomopathogenic bacteria *A. faecalis* non‐volatile compounds

#### Preparation of whole cell suspension and cell‐free supernatant

A single bacterial colony was transferred and grown in 100 ml TSY medium and shaken at 180 rpm for 24 h. The concentration of the whole cell suspension was adjusted to 3 × 10^6^ cells ml^−1^ using TSY medium (Wang *et al*., 2011).

In addition, to confirm that the antifungal effect we would see was not produced by random parts of the bacterial cell degradation, we also performed a cell‐free experiment. To prepare cell‐free supernatant, the whole cell suspension was centrifuged at 10 000 rpm for 10 min. The supernatant was filtered through a 0.22 mm filter, and the flow‐through was used as the cell‐free supernatant (Bussaman *et al*., 2012).

#### Petri dish experiment

First, the antifungal activity of *A. faecalis* non‐volatile compounds on *B. cinerea* and the three entomopathogenic fungi (*M. circinelloides*,* M. racemosus*,* R. variabilis*) was assessed using a modified method from (Bach *et al*., 2016). Briefly, 0.1 ml *A. faecalis* bacterial suspension (3 × 10^6^ cells ml^−1^) was smeared on TSY‐agar medium in 9 cm Petri dish, after which, a 5 mm wide fungal colony was inoculated in the centre of the Petri dish and incubated at 27°C in the dark (Wang *et al*., 2011). The control treatment consisted of the same manipulation but without *A. faecalis* in the substrate (*n* = 6 replicates per treatment and per fungal species, and repeated twice). Second, the antifungal activity of *A. faecalis* cell‐free supernatant on *B. cinerea* and the three entomopathogenic fungi was also evaluated. Briefly, *A. faecalis* cell‐free supernatant was added into TSY‐agar medium in 9 cm Petri dish at the concentration of 1%, 5% and 10%, after which, a 5 mm wide fungal colony was inoculated in the centre of the Petri dish and incubated at 27°C in the dark. The control treatment consisted of the same manipulation but without cell‐free supernatant in the substrate (*n* = 6 replicates per treatment and per fungal species, and repeated twice). For both experiments, after 5 days, the diameter of the fungal colonies was measured using the cross method. The effect of EPB on fungal colony size was assessed using a two‐way mixed effect ANOVA model, with fungal species and bacterial treatments as fixed factors, and repetition of the experiments as random factor (function lmer in the package lme4 (Bates *et al*., 2014) in R (R Development Core Team, 2015).

#### Cherry tomato fruits experiment

We next evaluated the effect of entomopathogenic bacteria *A. faecalis* (EPB) non‐volatile metabolites on plant‐pathogenic fungi *B. cinerea* (PPF) when growing on fresh cherry tomato fruits (*Lycopersivon esculentum* Mill). The EPB *A. faecalis* inoculum was prepared in 100 ml TSY medium on a rotary shaker at 180 rpm, 27°C for 24 h, and the bacterial suspension was diluted to 6 × 10^6^ cells ml^−1^. The PPF *B. cinerea* was cultured in PDA medium at 27°C in the dark. Before the experiment, spores were rinsed with 1 ml of sterile distilled water and diluted to 6 × 10^6^ cells ml^−1^. EPB and PPF suspensions were mixed at the rate of 1:1 (v/v) so that the EPB and PPF spore were both at 3 × 10^6^ cells ml^−1^ in the experimental mixture. For the control treatment, without PPF, the individual microorganism suspensions were diluted to 3 × 10^6^ cells ml^−1^ in TSY medium (for EPB), or in distilled water (for PPF). Before the onset of the experiment, the surface of cherry tomato fruit was sterilized by 75% ethanol and artificially wounded by piercing once with a 10 μl sterile pipette tip to a depth of about 3 mm. After that, 10 μl of 6 different suspensions were pipetted to the artificial wounded area, including (i) PPF spore suspension only, (ii) PPF spore diluted in TSY medium, (iii) EPB suspension only, (iv) the mixture of PPF and EPB, (v) TSY medium only and (vi) sterile distilled water only. The experimental design is shown in Fig. S2. The treated tomatoes were placed in a sterile plastic box (15 × 6 × 9 cm) sealed with Parafilm at 27°C in dark (*N* = 6 replicates per treatment, and repeated twice). Seven days after inoculation, the severity of infections was determined as the proportion of decayed area according to a scale from 0–4: 0 = No decay; 1 = decayed area < 1/8 of the total fruit area; 2 = decayed area between 1/8 to 1/4 of the total fruit area; 3 = decayed area between 1/4 to 1/2 of the total fruit area; and 4 = decayed area > 1/2 of the total fruit area. The PPF damage size on tomato fruits was assessed with two‐way mixed effect model, with fungal species and bacterial treatments as fixed factors, and experiment as random factor, as described above. As mentioned above, we again evaluated the EPB non‐volatile metabolites antifungal activity on PPF using cell‐free supernatant. The experimental was reproduced as explained above for the bacterial suspension experiment. The only difference consisted in replacing the bacterial suspension with the cell‐free supernatant. Final PPF spore was at 3 × 10^6^ cells ml^−1^ in the experimental mixture.

#### In vivo antifungal effects of EPB against EPF on G. mellonella larvae

The entomopathogenic fungi *M. circinelloides*,* M. racemosus* and *R. variabilis* were used for evaluating the antifungal effect of the EPB (*A. faecalis*) in vivo. For causing fungal infection, the pleopod of healthy larvae of *G. mellonella*, sterilized by 75% ethanol, was pierced by a 10 μl micro‐syringe and injected with 2 μl of EPB suspension in either TSY medium or sterile distilled water (Fig. S3). After that, larvae were placed into a sterile Petri dish (diameter 9 cm) and cultured at 27°C in the dark. Twenty‐four hours later, 100 μl of EPF spore suspension (3 × 10^6^ cells ml^−1^) larvae was dripped on the surface of the treated *G. mellonella* larvae, and immediately put on the PDA medium without any loss of spore suspension and cultured at 27°C in the dark. After 4 days, the number of *G. mellonella* infected by EPF was counted. Overall, the experiment included 12 treatments (Fig. S3); three EPF species plus one control treatment by three types of suspensions for inoculum (EPB, TSY medium, and sterile distilled water SDW). Each treatment had six replicates with one larva per replicate, and the experiment was performed twice. The same procedure was performed by replacing the bacterial suspension with the cell‐free supernatant. Final EPF spore was at 3 × 10^6^ cells ml^−1^ in the experimental mixture.

### Antifungal effect of entomopathogenic bacteria volatile organic compounds

#### Petri dish experiment

To measure the antagonistic effect of *A. faecalis* VOC emissions on plant‐pathogenic fungi *B. cinerea* colony growth, we built an experimental arena using 15 cm diameter Petri dishes. Inside each larger Petri dish, three lids of 6 cm diameter Petri dishes were evenly placed, one lid with TSY‐agar medium for EPB inoculation, one with PDA medium for fungal colony inoculation and one empty or with charcoal (Fig. S4). Three treatments for each repetition were (i) one large Petri dish containing fungal colonies only, (ii) one large Petri dish containing both the fungal colonies and the bacterial colonies and (iii) one large Petri dish containing the fungal colonies and the bacterial colonies, plus the third small dish containing charcoal. The charcoal has strong VOCs trapping properties, and we therefore expected that placing charcoal in the chamber would inhibit the putative effect of VOCs on fungal growth. The initial colony diameter of bacteria and fungi was 5 mm in all cases. After inoculation, the plates were immediately wrapped in Parafilm to maintain the VOCs inside the experimental chamber, and incubated at 22°C in the dark (Fig. S2). After 4 days, the diameter of the fungal colony on each lid was measured. Each treatment had six replications, and the experiment was repeated twice. The effect of EPB VOCs on fungal colony size was assessed with two‐way mixed effect model, with fungal species and bacterial treatments as fixed factors, and experiment as random factor, as described above.

#### Cherry tomato fruit experiment

The antifungal effect of VOCs released by EPB on PPF growing on tomato fruits was evaluated in sterile plastic boxes (15 × 6 × 9 cm; Fig. S5). In each box, on one side of each box, we placed six tomato fruits. The surface of cherry tomato fruit was sterilized by 75% ethanol and artificial wounded by piercing once with a 10 μl sterile pipette tip to a depth of about 3 mm. After that, the wound was smeared with PPF spore suspension, or sterile distilled water as control treatment. The other side of the plastic box contained a 35 mm diameter Petri dish filled with 10 μl of EPB (*A. faecalis*) suspension on TSY‐agar medium, or TSY medium only or no medium as controls. The density of EPB and PPF spore suspension was 3 × 10^6^ cells ml^−1^. In total, we thus obtained six treatments as follows: (i) PPF (on fruit) + EPB suspension on TSY‐agar medium (in Petri dish), (ii) PPF(on fruit) + TSY‐agar medium (in Petri dish), (iii) PPF (on fruit) + empty Petri dishes, (iv) sterile distilled water(on fruit) + EPB suspension on TSY‐agar medium (in Petri dish), (v) sterile distilled water (on fruit) + TSY‐agar medium (in Petri dish) and (vi) sterile distilled water (on fruit) + empty Petri dishes. After 6 days, the disease index was scored as described above. The effect of EPB VOCs on disease severity was assessed with one‐way mixed effect model, with the six treatments as fixed factors, and experiment as random factor, as described above.

#### EPB VOCs collection and analysis

Volatile organic compounds produced by the symbiotic bacteria were collected using a slightly modified method (Wgd *et al*., 2005). The colony of bacteria (diameter 5 mm) was inoculated into a 100 ml conical flask containing 10 ml TSY‐agar medium, and 1 ml of the internal standard solution (1 μg ml^−1^ of 1,4‐cineol, CAS No. CAS 470‐67‐7, Sigma‐Aldrich, dissolved in water). The flasks was next sealed and cultured 2 days at 22°C in the dark. Flasks with TSY medium only were used as control. Two days after symbiotic bacteria growth, VOCs were collected with solid phase micro‐extraction method (SPME). Before the collection of volatiles, the SPME fibres (Supelco, Bellefonte, PA, USA) were preconditioned in the GC injection port at 280°C for 10 min. One SPME fibre was next inserted in the flasks through the septum and exposed for 60 min to the headspace of the culture for VOCs collection. Five types of SPME fibres were employed to evaluate the extraction efficiency of the different polymers; including 75 μm Carboxen/PDMS, 7 μm PDMS, 100 μm PDMS, 65 μm PDMS/DVB and 50/30 μm DVB/CAR/PDMS (Fig. S6). VOCs were analysed with gas chromatograph–mass spectrometry (GC‐MS; Agilent 7890‐5975C Santa Clara, CA, USA). SPME fibres were next thermally desorbed in the injection port of the GC (Agilent 7890) by heating the fibre for 5 min at 250°C. VOCs separation was done on a HP‐5 ms non‐polar column (Agilent, 30 m × 0.25 mm i.d, 0.25 μm thin layer). Helium was used as carrier gas with a flow rate of 1.0 ml min^−1^. Manual injection was carried out in splitless mode. The temperature program worked as follows: 40°C for 5 min, 4°C min^−1^ to 100°C for 3 min, 5°C min^−1^ to 200°C for 3 min, 5°C min^−1^ to 280°C for 15 min. Detection was done on a MS (Agilent 5975C). Compounds were initially identifies by comparison with spectra from the NIST library and identification of the described VOCs (see Table 1) was confirmed using pure standards. Quantification of VOCs was done by calculating the ratio of individual compound peak area with the total peak area relatively and corrected by the area of the IS.

#### Antifungal effects of pure compounds VOCs against PPF and EPF

Based on the above VOCs analyses (see Results, Fig. 3, and Table 1) we selected several EPBs‐produced compounds for assessing specificity of VOCs antifungal effect. Dimethyl disulfide (2 μg ml^−1^), isobutyl isovalerate (0.4 μg ml^−1^) and isopentyl isopentanoate (0.1 μg ml^−1^) were three of the most abundant constituents present in the VOCs produced after 1 h exposure (Fig. S6). The pure compounds were purchased (Tokyo Chemical Industry Co., Ltd., Tokyo, Japan) and used for antifungal bioassays. For each single pure compound, we performed a serial dilution (10^2^, 10^3^, 10^4^, 10^5^, 10^6^) to obtain the corresponding concentration for antifungal bioassay (For dimethyl disulfide, the concentrations were 113, 11.3, 1.13, 1.13 × 10^−1^, 1.13 × 10^−2^ μmol ml^−1^, respectively; For isopentyl isopentanoate, the concentrations were 50, 5.0, 0.50, 0.50 × 10^−1^, 0.50 ×10^−2^ μmol ml^−1^, respectively; For isobutyl isovalerate, the concentrations were 54, 5.4, 0.54, 0.54 × 10^−1^, 0.54 × 10^−2^ μmol ml^−1^, respectively). For assessing the effect of the three compounds’ mixture of three volatile organic compounds, dimethyl disulfide, isobutyl isovalerate and isopentyl isopentanoate were mixed according to the ratio of 20:4:1 (m:m:m), which is based on the SPME results, and then diluted (10^2^, 10^3^, 10^4^, 10^5^, 10^6^) for bioassay. One hundred microlitres of single compounds or the mixture of the three compounds for each dilution was added to sterile filter paper discs (5 mm) and placed on PDA medium for evaluating the antifungal effects on PPF (*Botrytis cinerea*), or TSY‐agar medium for evaluating the antifungal effects on EPF *Mucor circinelloides*,* Mucor racemosus*,* Rhizomucor variabilis* in divided agar plates (Fig. S7). After 4 days, the diameter of the fungal colonies was measured. Control Petri dishes only had the corresponding fungi. Each treatment had six replications and the experiment was performed twice. The effect of VOCs on fungal colony size was assessed with two‐way mixed effect model, with fungal species and serial dilution treatments as fixed factors, and experiment as random factor, as described above for the DMDS and the mixture experiment separately.

## Conflict of interests

None declared.

## Supporting information


**Fig. S1**. Overview of the experimental design for measuring the impact of EPNs‐associated bacteria on entomopathogenic fungi and plant pathogenic fungi.
**Fig. S2.** Experimental design for testing the antifungal effects of EPB metabolites against PPF on cherry tomatoes fruits.
**Fig. S3**. *In vitro* inhibition of EPF growth on *G. mellonella* larvae by *EPB Alcaligenes faecalis* inoculation
**.Fig. S4.** Experimental design for testing the antagonistic effect of EPB VOCs on PPF or EPF.
**Figure S5. .** Experimental design for testing the effects of EPB VOCs against PPF on cherry tomatoes fruits.**Fig. S6.** Total ion chromatograms for EPB VOCs emissions.
**Fig. S7**. Experimental design for testing the antifungal effects of pure volatile organic compounds (VOCs) against PPF and EPF.
**Fig. S8**. Antifungal activity of isobutyl isovalerate and isopentyl isopentanoate against PPF *Botrytis cinerea* (A,B), and EPF *Mucor circinelloides* (C,D), *M. racemosus* (E,F), and *Rhizomucor variabilis* (G). Both of isobutyl isovalerate and isopentyl isopentanoate didn't show any antifungal activity to the PPF and EPF.
**Appendix S1** ***.*** PCR amplifications, cloning and analyses.
