# Supplementary material for: The symbiotic bacteria Alcaligenes faecalis of the entomopathogenic nematodes Oscheius spp. exhibit potential biocontrol of plant‐ and entomopathogenic fungi
Source: Microb Biotechnol. 2019 Jan 7;12(3):459–71. doi: 10.1111/1751-7915.13365 (PMC6465237; doi:10.1111/1751-7915.13365)
Supplement: Supplementary file 1 — Fig. S1. Overview of the experimental design for measuring the impact of EPNs‐associated bacteria on entomopathogenic fungi and plant pathogenic fungi. Fig. S2. Experimental design for testing the antifungal effects of EPB metabolites against PPF on cherry tomatoes fruits. Fig. S3. In vitro inhibition of EPF growth on G. mellonella larvae by EPB Alcaligenes faecalis inoculation .Fig. S4. Experimental design for testing the antagonistic effect of EPB VOCs on PPF or EPF. Figure S5. . Experimental design for testing the effects of EPB VOCs against PPF on cherry tomatoes fruits.Fig. S6. Total ion chromatograms for EPB VOCs emissions. Fig. S7. Experimental design for testing the antifungal effects of pure volatile organic compounds (VOCs) against PPF and EPF. Fig. S8. Antifungal activity of isobutyl isovalerate and isopentyl isopentanoate against PPF Botrytis cinerea (A,B), and EPF Mucor circinelloides (C,D), M. racemosus (E,F), and Rhizomucor variabilis (G). Both of isobutyl isovalerate and isopentyl isopentanoate didn't show any antifungal activity to the PPF and EPF. Appendix S1 . PCR amplifications, cloning and analyses. [file MBT2-12-459-s001.docx]

Supplementary material – *Microbial Biotechnology*

**Generalized antifungal effect of symbiotic bacteria associated with soil-dwelling entomopathogenic nematodes**

Shaojie Shan, Wenwu Wang, Chunxu Song, Minggang Wang, Bingjiao Sun, Yang Li, Yaqi Fu, Xinghui Gu , Weibin Ruan, Sergio Rasmann

**Appendix S1*.*** **PCR amplifications, cloning and analyses**

For identification analyses in this study, ITS region was amplified from the genomic DNA of the nematodes and the fungi, respectively. 16S was amplified from the genomic DNA of bacteria. Each PCR reaction was carried out using a total volume of 25 µl containing 1 µl DNA template, 2.5 µl 10×PCR Buffer (containing MgCl_2_), 2 µl 0.2 mM of dNTPs, 0.5 µl 10 µM of each primer, 2.5 µl 1.5 units of Polymerase and 18.25 µl of ddH_2_O. The ITS primers for EPNs were ITS-F (5-TTG AAC CGG GTA AAA GTC G) and ITS-R (5-TTA GTT TCT TTT CCT CCG CT) ([Stock et al. 2001](#_ENREF_37)). The primers used for amplification of 16S ribosomal DNA (16S rDNA) sequences were 16S-27F (5-AGA GTT TGA TCC TGG CTC AG) and 16S-1492R (5-GGT TAC CTT GTT ACG ACT T). The ITS ribosomal DNA primers for fungi us were ITS1 (5-TCC GTA GGT GAA CCT GCG G) and ITS4 (5-TCC TCC GCT TAT TGA TAT GC). The amplifications were performed in a thermocycler with an initial denaturation step of 95 °C for 3 min, followed by 35 cycles of 95°C for 30 s, 55°C for 45 s, 72 °C for 75 s, and one cycle at 72 °C for 10 min. PCR products were electrophoresed in 1.5% agarose gel. PCR products were purified, cloned and sequenced in both directions sequenced by GENEWIZ Biotechnology Co., Ltd., Suzhou, China). Sequences were assembled and edited in BioEdit 7.2.5 ([Hall 1999](#_ENREF_12)). Sequences were finally subjected to Blast-searches using GenBank for taxonomic group assignments.


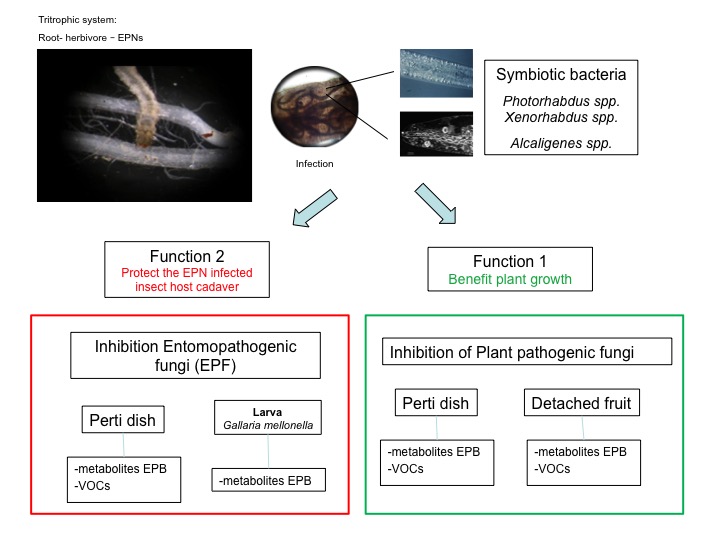


Figure S1. Overview of the experimental design for measuring the impact of EPNs-associated bacteria on entomopathogenic fungi and plant pathogenic fungi.


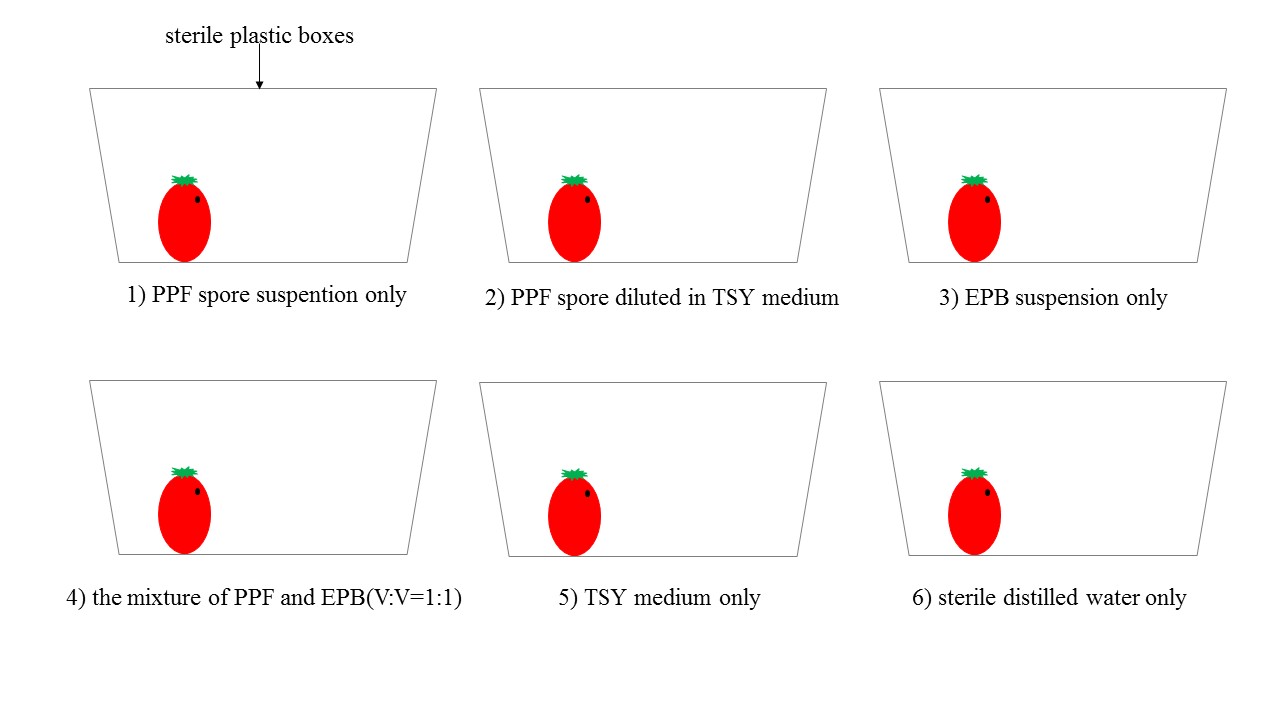


**Figure S2.** Experimental design for testing the antifungal effects of EPB metabolites against PPF on cherry tomatoes fruits. The surface of cherry tomato fruit was sterilized by 75% ethanol and artificial wounded by piercing once with a 10 μL sterile pipette tip to a depth of about 3mm. After that, 10 μL of 6 different suspensions were pipetted to the artificial wounded area, including; 1) PPF spore suspension only,, 2) PPF spore diluted in TSY medium, 3) EPB suspension only, 4) the mixture of PPF and EPB(V:V=1:1), 5) TSY medium only, and 6) sterile distilled water only. For all treatments included PPF, the final spore density was equal. The treated tomatoes were placed in a sterile plastic box (15 cm × 6cm × 9 cm) sealed with parafilm at 27 °C in dark. While here only one fruit per box is shown, during the experiment each plastic box contained 6 fruits.


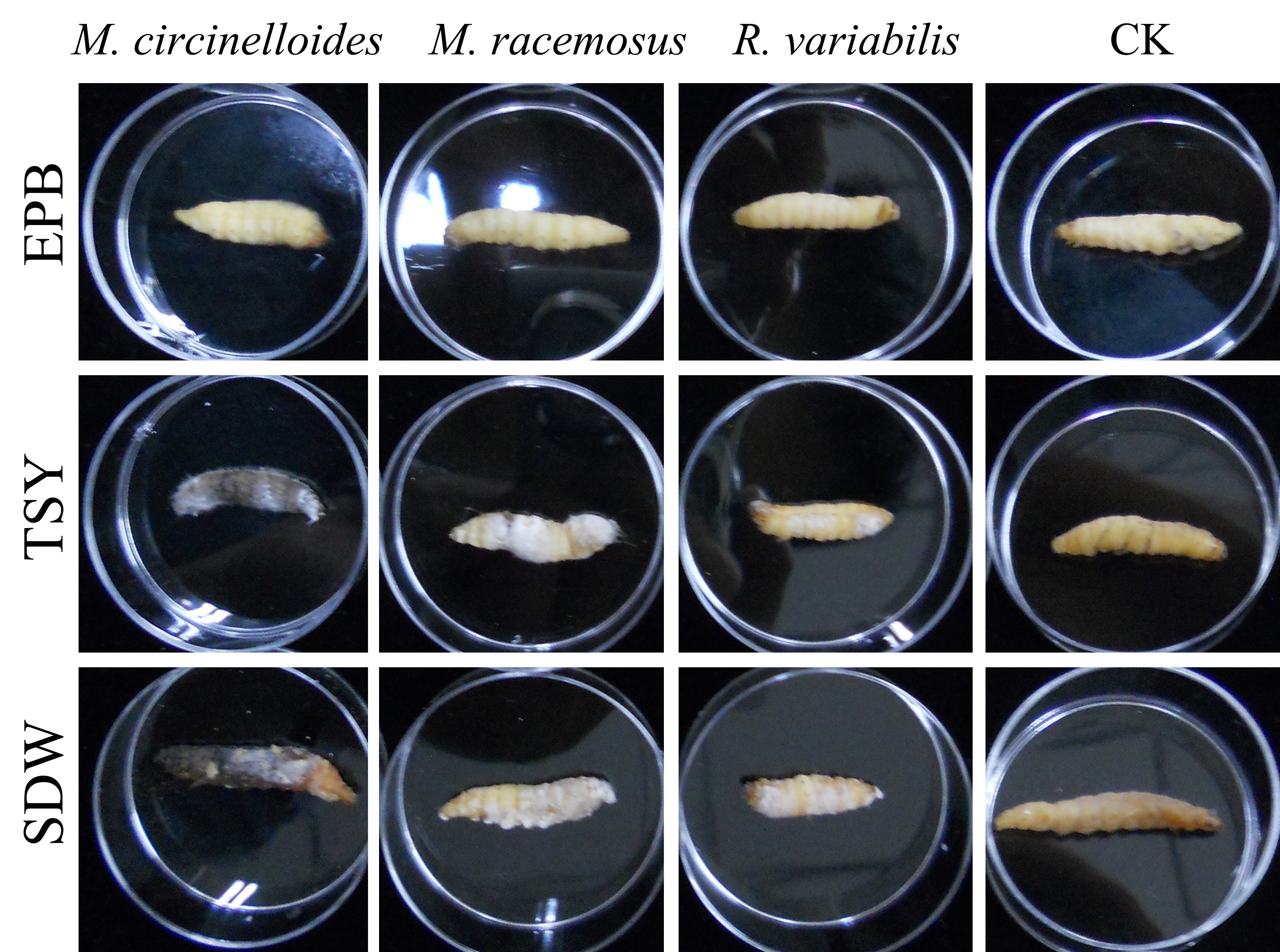


**Figure S3**. *In vitro* inhibition of EPF growth on *G. mellonella* larvae by *EPB Alcaligenes faecalis* inoculation**.** EPB: symbiotic bacteria; TSY: TSY medium; SDW: sterile distilled water. EPF: entomopathogenic fungi. Totally, it included 12 treatments, 9 combinations of three EPF and 3 types of inoculum (EPB, TSY, and SDW), three types of inoculum alone used as the corresponding control treatments. Each treatment had 6 replications with one larva for each and the experiment was performed twice. Three EPF species were included *Mucor circinelloides*, *M. racemosus*, *Rhizomucor variabilis*.


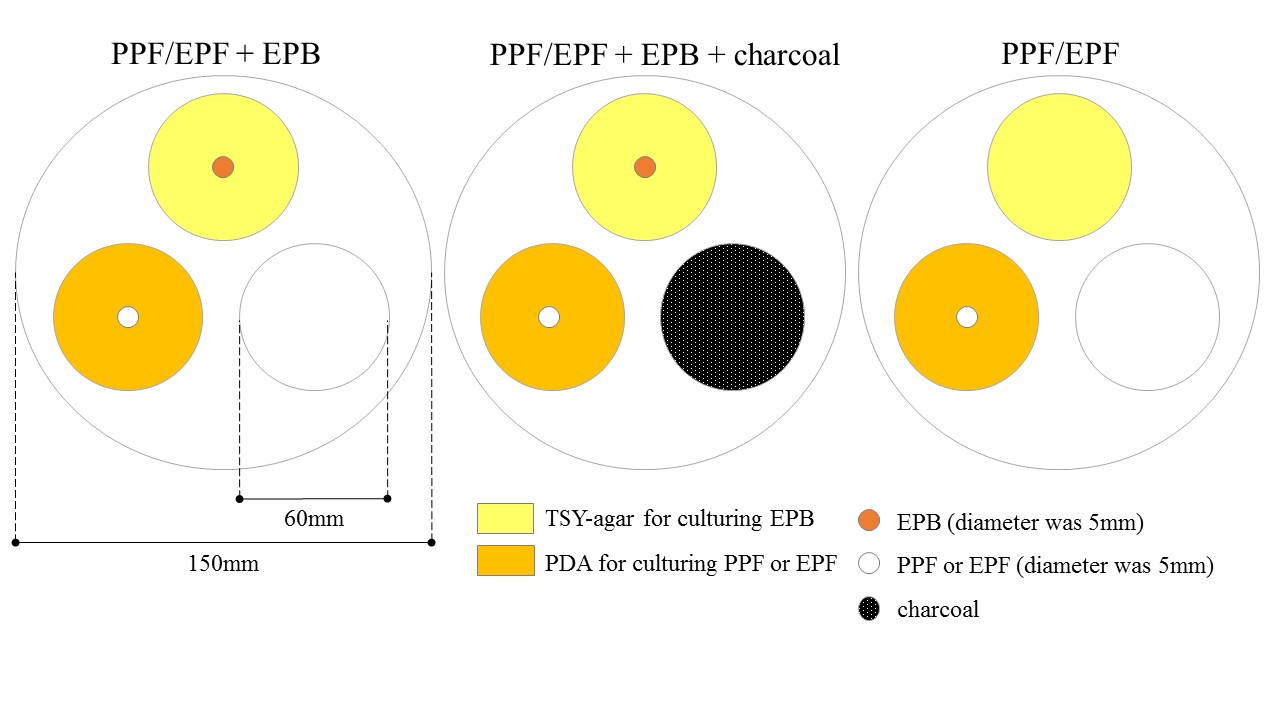


**Figure S4.** Experimental design for testing the antagonistic effect of EPB VOCs on PPF or EPF. Inside each 15 cm diameter Petri dishes, three lids of 6 cm diameter Perti dishes were placed evenly. One lid with TSY-agar medium for EPB inoculation, one with PDA medium for fungal colony inoculation, and one empty or with charcoal. Three treatments were included: 1) PPF/EPF only, 2) EPB+PPF/EPF, and 3) EPB+PPF/EPF +Charcoal. The initial colony diameter of symbiotic bacteria and PPF was 5mm. After inoculation, the plates were immediately wrapped in Parafilm to maintain the VOCs, and incubated at 22℃ in dark.


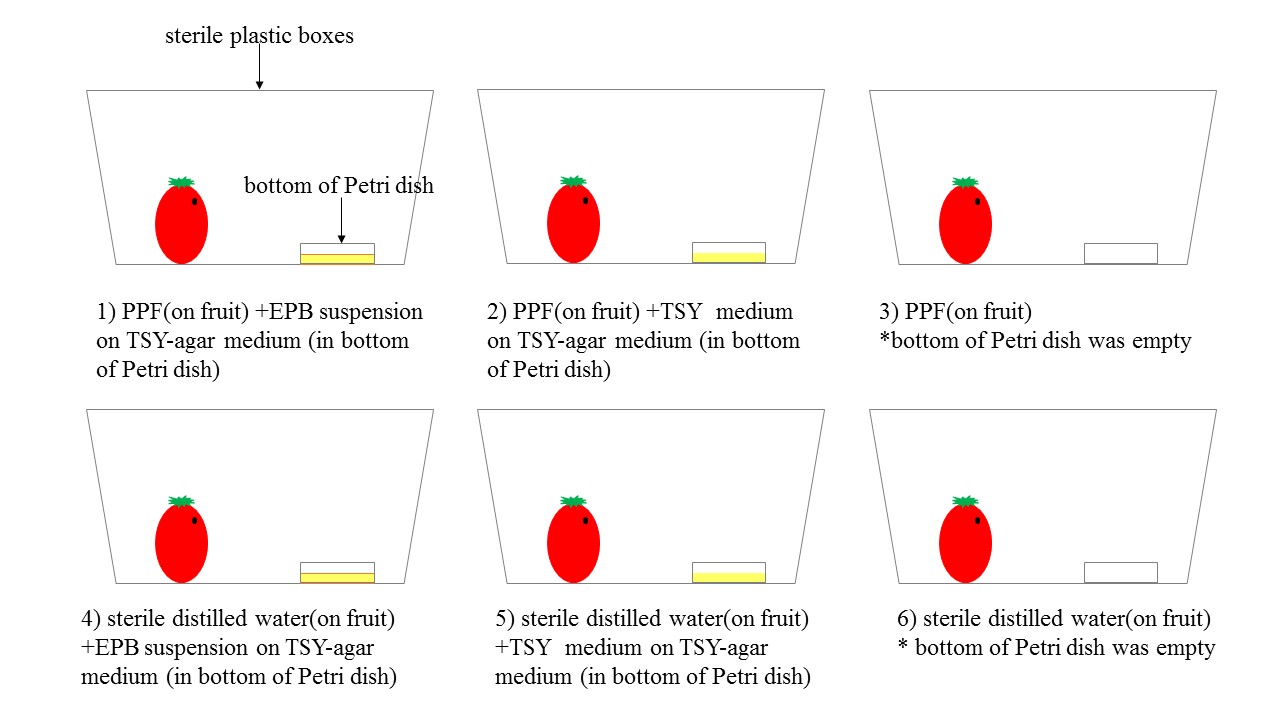


**Figure S5. .** Experimental design for testing the effects of EPB VOCs against PPF on cherry tomatoes fruits. The antifungal effect of VOCs released by EPB was evaluated in a sterile plastic boxes (15 cm × 6cm × 9 cm). In each box, on one side, there were 6 tomato fruits (graphic figure only showed one fruit), smearing with PPF spore suspension/sterile distilled water, and on the other side, a 35 mm dimeter Petri dish filled with 10μl EPB suspension (3 ×10^6^ cells/ml, on TSY-agar medium)/ TSY-agar medium/ empty. The density of PPF spore suspension was 3 ×10^6^ cells/ml. It consists of six treatments:1) PPF(fruit) +EPB on TSY-agar medium, 2) PPF(fruit) +TSY-agar medium, 3) PPF(fruit)+empty petri dishes, 4) sterile distilled water(fruit) + EPB on TSY-agar medium, 5) sterile distilled water(fruit)+ TSY-agar medium, 6) sterile distilled water(fruit) + empty petri dishes.


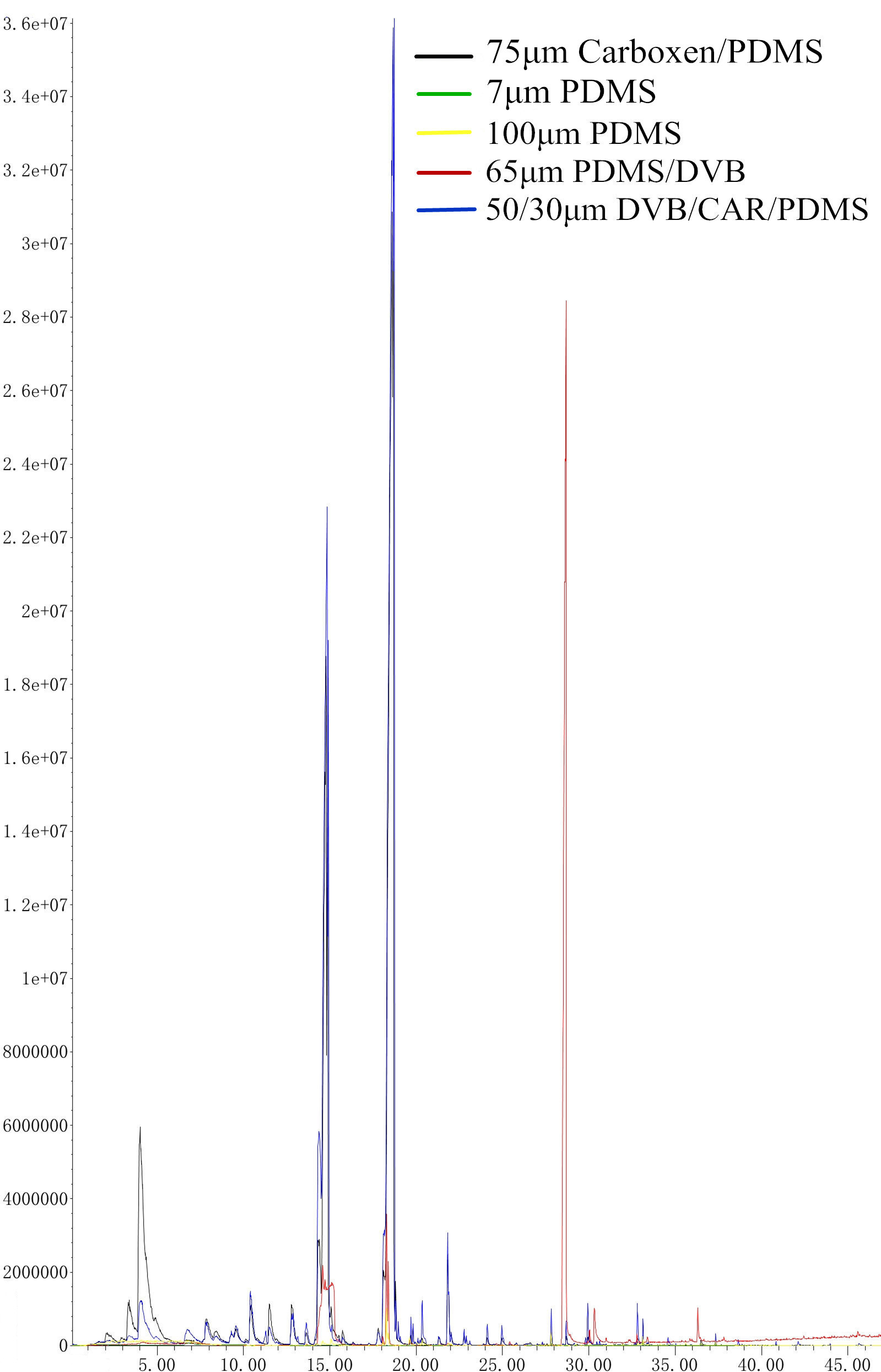


a)

**c**

**a**

**f**

**
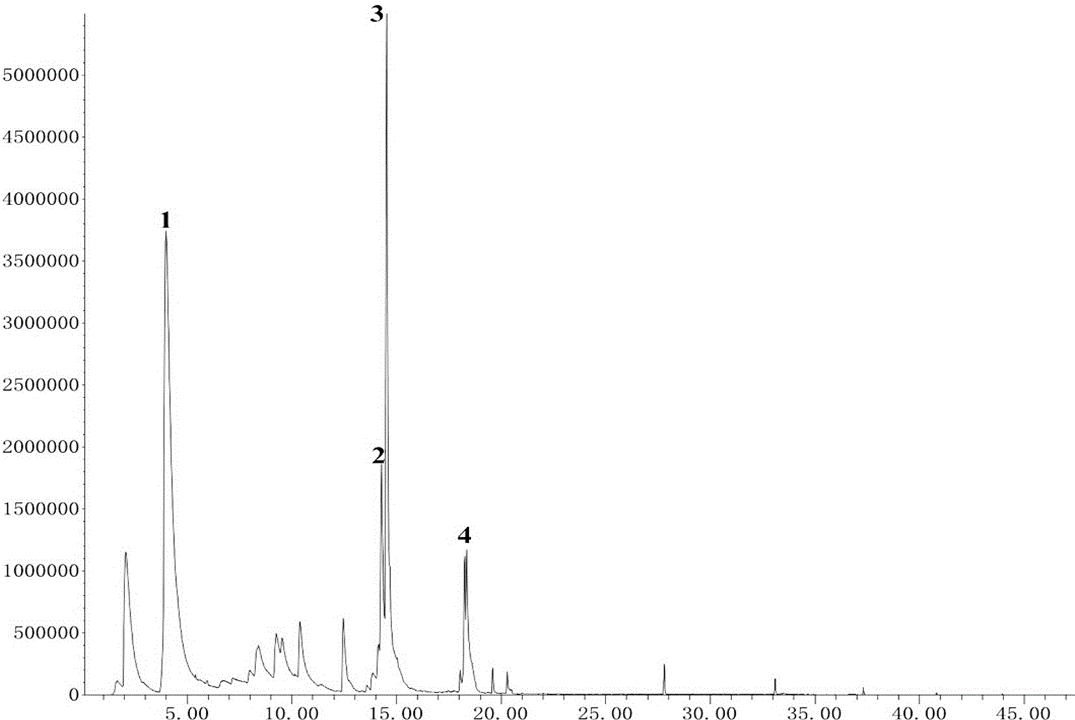
**

b)

**Figure S6.** Total ion chromatograms for EPB VOCs emissions. Panel a) shows five different chromatograms obtained using five different SPME fibers: 7μm PDMS and 100μm PDMS didn’t produce almost any peak. 65μm PDMS/DVB produced five obvious peaks. 75μm Carboxen /PDMS and 50/30μm DVB/CAR/PDMS produced the most complete chromatographs. Accordingly, we choose the 75μm Carboxen/PDMS as the optimal adsorption efficiency fiber. Panel b) shows the chromatogram obtained using fiber (75μm Carboxen/PDMS). The labelled peaks are as follows: 1, dimethyl disulfide; 2, isobutyl isovalerate; 3 internal standard 1,4-cineol; 4, isopentyl isopentanoate.


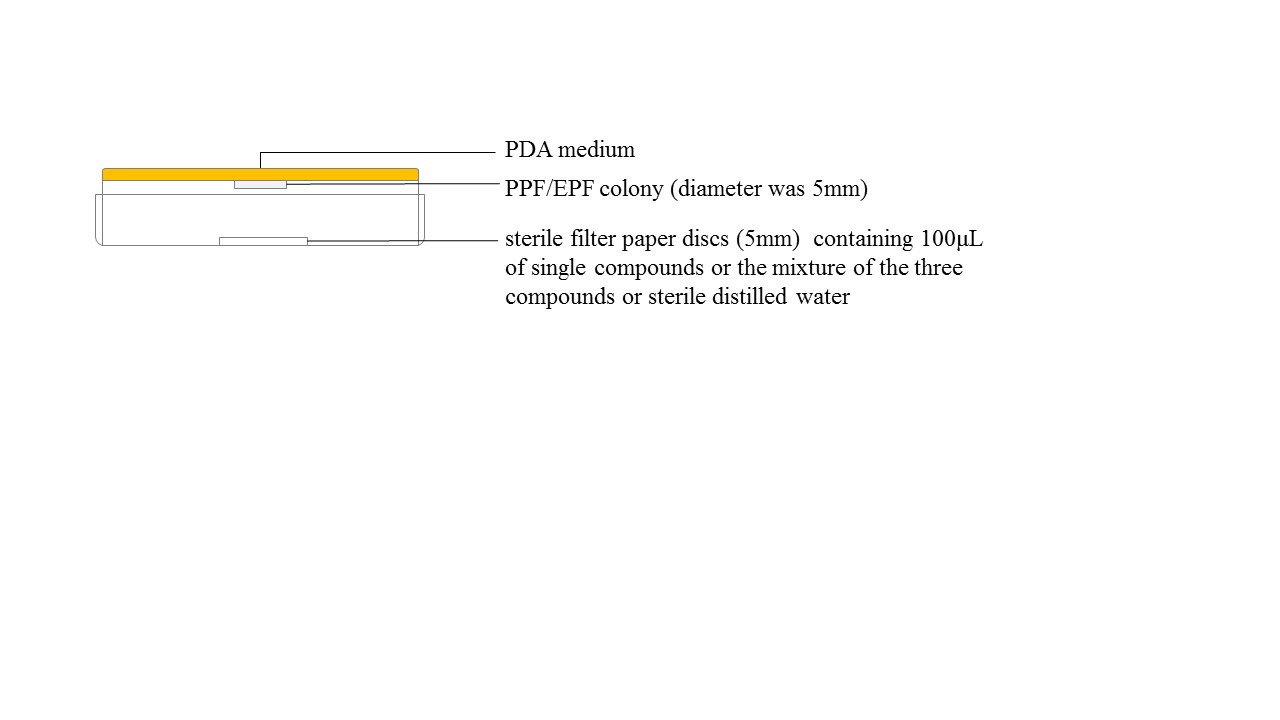


**Figure S7.** Experimental design for testing the antifungal effects of pure volatile organic compounds (VOCs) against PPF and EPF. One hundred microliters of single compounds or the mixture of the three compounds for each dilution was added to sterile filter paper discs (5mm) and placed on PDA medium for evaluating the antifungal effects on PPF (*Botrytis cinerea*)*,* or EPF *Mucor circinelloides*, *Mucor racemosus*, *Rhizomucor variabilis* in divided agar plates. Control Petri dishes added the sterile distilled water.
