# Supplementary material for: The symbiotic bacteria Alcaligenes faecalis of the entomopathogenic nematodes Oscheius spp. exhibit potential biocontrol of plant‐ and entomopathogenic fungi
Source: Microb Biotechnol. 2019 Jan 7;12(3):459–71. doi: 10.1111/1751-7915.13365 (PMC6465237; doi:10.1111/1751-7915.13365)
Supplement: Supplementary file 2 [file MBT2-12-459-s002.docx]

**A**

**B**

**C**

**E**

**D**

**F**

**G**

**Figure S8.** Antifungal activity of isobutyl isovalerate and isopentyl isopentanoate against PPF *Botrytis cinerea*(A,B)*,* and EPF *Mucor circinelloides*(C,D), *M. racemosus*(E,F), and *Rhizomucor variabilis*(G). Both of isobutyl isovalerate and isopentyl isopentanoate didn’t show any antifungal activity to the PPF and EPF.
